# Transmission of food literacy to youth in Norwegian childcare institutions – a qualitative study

**DOI:** 10.29219/fnr.v68.9803

**Published:** 2024-01-26

**Authors:** Marianne S. Morseth, Siyamali Gananathan, Sigrun Henjum, Laura Terragni

**Affiliations:** Department of Nursing and Health Promotion, Oslo Metropolitan University, Oslo, Norway

**Keywords:** food, meals, residential care, adolescents, youth, obesity, non-communicable diseases

## Abstract

**Background:**

Youth in childcare institutions may have lower levels of food literacy compared to other youth. Food literacy, indicating the ability to plan and prepare meals from scratch, is associated with consuming healthier diets.

**Objective:**

The objective of this study was to explore how food literacy is transmitted to youth through involvement and participation in food-related activities in Norwegian childcare institutions.

**Design:**

Data were collected through qualitative semi-structured interviews with 13 staff and 8 adolescents (>16 years of age) selected by convenient sampling in childcare institutions (*n* = 6) in South-East Norway.

**Results:**

We found that the institutional context provided both opportunities and limitations for transmitting food literacy. The staff tended to prefer a soft approach to transmitting food literacy due to the youth being perceived as vulnerable and a focus on youth autonomy. The youth showed ambivalent interests in participating in food-related activities and wished for their need to decide how much to be involved to be respected. A firm approach was perceived to increase the risk of conflict.

**Discussion:**

Our findings are best interpreted in light of a childcare institution being at the intersect between the homely and public spheres. There was ambivalence among staff between following institutional guidelines and caring for the different needs of residents. Food was often referred to as symbolizing something else in the relationship between staff and youth, such as care, structure, autonomy, and a normal family life.

**Conclusion:**

Initiatives aiming at including food literacy in care relations in childcare institutions are recommended.

## Popular scientific summary

- The institutional context provided both opportunities and limitations for the staff to transmit food literacy to the youth.
- Food was regarded as a token of care and cooking together as a way to build relationships.
- The staff preferred what may be called a soft approach to transmitting food literacy and the youth showed ambivalent interest in being included in food-related activities.
- Initiatives to improve food literacy in childcare institutions should consider care relations.

All children have a right to adequate nutrition, good health, and well-being (1). Over the past decades, there has been rising concern that changes in our food system prevent children and adolescents from consuming nutritious diets (2). Specifically, the nutrition transition has caused increased access to energy-dense, ultra-processed, nutrient-poor foods leading to increased prevalence of obesity and non-communicable diseases (NCDs), such as diabetes and cardiovascular disease (3). The combination of increased supply of ultra-processed foods (UPFs) and a constant exposure to messages promoting ‘healthy eating’ has caused confusion for those who must navigate the complex modern foodscape (4, 5). Physiological, cognitive, and psychological characteristics of adolescents make them vulnerable to features across the food system that result in suboptimal nutritional outcomes (6). The rise in consumption of UPFs has further been coupled with a decline in food-related activities such as family meal planning and cooking (7, 8), and a ‘deskilling’ when it comes to food and nutrition (9), indicating reduced food literacy. According to Vidgen and Gallegos, food literacy is a collection of interrelated knowledge and skills enabling preparation and consumption of foods that meet dietary needs (10). Food literacy is acquired across the lifespan (11), and the family is the core environment where children build their skills, preferences, and routines (7, 8).

Food literacy is required by adolescents as they transcend into adulthood so they may navigate complex food environments, develop a positive relationship with food, and reduce the risk of obesity and NCDs (12). The framework for food literacy proposed by Vidgen and Gallegos consists of four separate dimensions: to plan and manage, select, prepare, and eat foods that meet nutritional needs. In this framework, plan and management refers to, for example, prioritizing money and time for food and making feasible food decisions that balance food needs with available resources. Selection implies, for example, accessing food through multiple sources and determining what is in a food product. Preparing refers to making a good tasting meal from whatever food is available and applying basic principles for safe food hygiene and handling. Finally, eating indicates, for example, understanding how balancing food may have an impact on personal well-being and being able to join in and eat in a social way (10). Food literacy is linked to improved dietary practices (13), which once acquired during childhood and adolescence tend to track into early adulthood (14). Improving food literacy by creating food learning opportunities prior to transition into adulthood may, thus, lead to healthier dietary behaviors.

Low levels of food literacy are particularly concerning among vulnerable young people (15). One such group is young persons who live in childcare institutions. Residence in childcare institutions in Norway is mainly for young persons of 13–19 of age, while younger children are normally placed in foster care (16). The most common reasons for young people being placed in childcare institutions are behavioral problems, neglect, or drug use. Childcare institutions in Norway are run by governmental bodies such as municipalities, non-governmental organizations (NGOs), and private actors (17), all funded by the government. The staff in these institutions will have roles as caretakers helping the young persons with matters related to school, healthcare, meals, and so on (16). One main aim is to prepare young people for an independent life after leaving the institution (18). By the end of 2022, 340 young people between the ages of 13 and 17 lived in childcare institutions nationally (19).

In Norway, young persons who live in childcare institutions are entitled to have their basic needs met. For instance, they have the right to sufficient food of good quality and a nutritionally adequate diet, which lays the foundation for good health and an active life (20). Norwegian childcare institutions should comply with national dietary guidelines (21). It is the shared responsibility of all staff to promote healthy diets; however, a previous study indicates that few have formal competence in food and nutrition (22). At the same time, food should not only be viewed according to its health-promoting properties but also carries symbolic meaning. A study investigating the role of food in childcare institutions in Scotland viewed food as creating a sense of belonging, negotiating relationships, learning social skills, and expressing feelings (23). Food and meals are arenas where staff may show care and consideration, and the young people may nurture relations in a homely atmosphere (16, 24). Meals also bring routine and structure to the youths’ lives that may have been lacking previously (16). A study from 2017 showed that staff at childcare institutions in Eastern Norway attributed different values to food and meals and experienced that motivating youth to make healthier food choices was challenging (22). In family settings, the food choices of children and adolescents result from a balance between exertions of autonomy and parental control mechanisms, which are expected to change over time (25). While previous studies have investigated the transmission of food literacy in the family setting, little is known about the experiences, processes, and potential tensions in dealing with transmitting knowledge of food and nutrition in childcare institutions.

The objective of our study was to explore through qualitative interviews how food literacy is transmitted to young persons aged 13–19 years through involvement and participation in food-related activities in Norwegian childcare institutions.

## Materials and methods

The data collection took place in 6 out of 7 childcare institutions run by a non-profit organization in South-East Norway with 300 full-time staff and 80 residents 13–19 years of age. All of the institutions provided separate apartments for the older residents. Some of the institutions had a designated staff member responsible for the food service, but there were no formal requirements for holding this position. Any staff member would go to the grocery store on specific weekdays, while some also ordered home delivery of groceries. All meals were served or prepared at the institution, and although the intention was that young people who had moved to their own apartment should train to plan for and prepare their own meals, they could also attend meals at the institution. Finally, all residents received a small weekly allowance that they could spend freely.

This study has a qualitative research design with interviews both with staff working in the institutions and adolescents residing there. This study was part of a ‘needs assessment’ of a larger project aimed at promoting food literacy in the childcare institutions, as requested by the non-profit organization. Informants were included through convenience sampling (26). We aimed at including not only staff who had daily contact with the young people but also staff working shorter hours or during weekends as we wanted to grasp different perspectives and experiences. A reference group was established at the beginning of the project to provide guidance, facilitate communication, and monitor and discuss progress in the projects. Staff and residents were informed about the project and recruited by the leader at their respective workplace. An initial e-mail describing the project was sent to the leaders. Staff were informed about the project and asked during institutional staff meetings whether they would like to participate. A list of staff and young persons who wished to participate was then forwarded to the researcher in charge of conducting the interviews. The potential participants received a consent form by e-mail that needed to be returned before the interview. The participants’ age should be 16 years or older to participate in this study. In total, 13 staff members and 8 young people aged 16–19 years were included. Among the youths, four lived in their own apartments provided by the childcare organization. The data collection was conducted from September to December 2020. Researchers had to sign a confidentiality statement at the beginning of the project.

For staff, individual in-depth interviews were conducted using a semi-structured interview guide based on the food literacy model proposed by Vidgen and Gallegos and focusing on experiences with meals and youth involvement in the institutions. The interview guide was pilot tested twice in the presence of three female researchers: EW (MSc in Public Health Nutrition [PHN]), SG (Master’s student in PHN), and LT. The results from the pilot tests were later included in this study. All subsequent interviews were conducted by one of the mentioned researchers. Interviews with staff were recorded using a voice recorder and lasted from 46 to 144 min (average 86 min). Interviews with young persons were also individual and based on a semi-structured interview and focused on aspects related to food literacy, such as involvement in food-related activities, reflections regarding meals, ‘healthy food’ versus ‘food for enjoyment’, different needs, and, finally, competencies and need for measures to improve food literacy. The young persons were asked if they preferred to be alone or in the presence of a staff member, and all youths – with one exception – preferred to have a staff member present. The interviewers introduced themselves by name, role, and field of study; no other relationship was established prior to the data collection. The interviews with youths lasted between 18 and 60 min. Interviews were recorded and later transcribed verbatim using f4transcript (Audiotranskription, Marburg, DE), and non-verbal cues (such as laughter and pauses) were included.

The data were analyzed using thematical analysis, as described by Braun and Clark (27), combining a deductive and inductive approach. Interviews were read and coded, searching for themes related to the food literacy model by Vidgen and Gallegos (10). The coding process of the interviews with the staff was conducted by SG and LT, while EW, MA, and LT coded the interviews with the residents. Three interviews from each subgroup were analyzed independently by research team members to achieve agreement on interpretations and code structure. The interviews of the two subgroups, staff and young persons, were then analyzed by SG and MA, respectively, under the supervision of LT. Preliminary coding was presented and discussed at common meetings with the reference group. Interviews were reread, and the coding was revised by MSM and LT during the drafting of the article. Data were coded using NVivo12 (QSR International Pty Ltd., MA, USA).

Due to the COVID-19 pandemic, all interviews were conducted using Microsoft Teams. The study was planned, and the results presented according to the ISSM-COREQ checklist for qualitative research. Ethical approval was obtained from the Norwegian Center for Research Data (NSD; ref. 436924), and an informed consent was obtained from all participants. Personal data were anonymized and kept separate. The audio files were stored on an encrypted device and erased after the data had been transcribed.

## Results

### Informants

Thirteen staff members, of which three were men, from six institutions were included. The informants worked different shifts, and the length of employment varied (Table 1). Three informants were in charge of the food service at the time of the interview, two had been in this position previously, and one was about to get this role.

**Table 1:** Information about the staff informants interviewed

Informant	Gender	Length of employment (years)	In charge of the food service^a^
1	Female	Less than 1	Yes
2	Female	Less than 1	Yes
3	Female	No data	Yes
4	Female	1	No
5	Male	6	No
6	Female	2	No
7	Male	20	No
8	Female	10	No
9	Female	12	No
10	Female	11	No
11	Female	2	No
12	Female	6	No
13	Male	3	No

a Role includes planning of dinner menu and for some grocery shopping.

Eight young people (four boys and four girls, aged 16–19 years) were interviewed.

### Food literacy

#### Plan and manage

With regard to the youths’ involvement in planning food intake and making food decisions, all staff informants expressed that the young persons had the opportunity to influence menu planning through participation in weekly house meetings. In particular, the menus for Friday and Saturday were left open to incorporate the young people’s preferences. Participation in house meetings was mentioned by one of the staff as an important learning arena:

… Has a lot of social learning, to sit together and make suggestions and plan what everyone should eat for the rest of the week. (staff informant 14)

Several staff informants were cognizant that involvement in menu planning in many instances also increased participation in food preparation and shared meals. However, attendance at house meetings was mentioned by some to be low, and in one institution, snack foods were served in the meetings to induce participation. Several of the staff reported that the young people were not always engaged in making suggestions, in which case the staff would set the menu:

They do not always come up with suggestions (…). If they don’t come up with suggestions, the staff will set the menu. (staff informant 10)

It is the staff that make the meals here. So, they make the food for me. Sometimes I tell them: ‘just buy something’. Other times, I tell them what I want. (youth informant 3)

Both staff and youth informants mentioned that the topic ‘fish for dinner’ was discussed during menu planning. Commonly, staff referred to institutional guidelines where fish was to be served twice a week, and some stated that this was also accepted among the residents, while one of the youth informants expressed that they would try to propose something else. Several staff informants felt that although user involvement was important, a balance needed to be kept during menu planning. Interestingly, the staff’s view of the young people’s role varied. One staff informant referred to their requests as reasonable:

In general they come up with good and reasonable suggestions which we feel are … ok. We absolutely try to accommodate to … their wishes. (staff informant 12)

Another staff informant voiced more concern:

… so they may come up with ideas, but … eh … a lot of it we, in a way, end up deciding. If not, the result may be a lot of junk. (staff informant 4)

In relation to this, one staff informant explained that they would keep a shopping list in the common area for everyone’s use. Items on the list that were deemed unsuitable were erased, followed by an explanation to the young person who had written down the item. Economy was also used in some instances to try to steer the youths in a healthier direction. For instance, one staff informant explained that they would not put frozen pizza on the institution’s shopping list, so in the event, this was strongly desired, and the young person had to spend their own pocket money. The weekend menu most influenced by the youths typically consisted of homemade pizza, lasagna, hamburgers, and tacos.

(…) during the weekend we often end up with tacos on Friday and pizza on Saturday. That is how it is. (staff informant 1)

Another area where the young people could enhance their ability to plan food intake was by preparing their own packed lunches for school. While the staff informants were aware that this was a task the young persons could fully perform, it was not a requirement, and some felt that providing a packed lunch was associated with care:

Ehm … they get help if they want it, and sometimes we do it, not because they don’t know how, but because there is eh … a lot of care in doing it and … helping out with packed lunches, kind of doing a bit extra. (staff informant 12)

#### Select

Selection of food includes aspects related to availability and making choices. In general, the staff appeared to take a soft approach to talking about food and health or influencing the young people to select healthy foods. One arena where the youths could actively take part in selecting foods was at the grocery store, but relatively few went along for grocery shopping with the staff. This was due to structural reasons, such as home delivery of groceries and much of the shopping occurring during the day when the young people were in school or busy with extracurricular activities. Grocery shopping could also be perceived to be a stressful experience for the youths:

But sometimes they think it’s a bit stressful because there is so much, we have to buy. We go with two full shopping baskets, so they think it’s a bit tiring to be part of that shopping round (…). (staff informant 13)

Some staff informants suspected that some of the young people came to the store to try and influence what would be served in the common area, and often in an unhealthier direction. One staff informant specifically mentioned that they used the trip to the store to actively teach young people about the content of different foods and how to read food labels:

(…) we do talk about it when we go to the store, it is a … a nice opportunity if there are kids with us at the store; they learn to read at the back of products to find what is here (…). In addition to thinking about what is affordable, I try to make them think about what is healthy and which products they should choose (…). (staff informant 13)

During the interviews with the young persons, it appeared that they had little experience with planning and shopping for groceries, and some were interested in learning more. Those who lived in their own apartment had more experience with food shopping and planning than those still living in the institutions. One recalled that the first time he shopped for food was shocking:

How expensive it is! [to buy food]. I was kind of shocked. (youth informant 2)

A gradual increase in demands for participation in food-related activities, the closer the young individuals came to living by themselves, which was also expressed by staff:

… when she (name of colleague) started with this dietary (…) she had a kind of project with different youths, and then they became a lot more conscious and, those youths were 18 and 19 years old and (…) were more aware and were about to move into a flat. The last half year before they move out you work more intensely with them, when they are, like, almost grown up. (staff informant 13)

The staff’s behavior in relation to promoting healthy eating may be understood as a form of nudging or gently pushing the young people to select healthier foods. Some staff would increase accessibility and potential intake of fruit and vegetables by serving freshly cut vegetables when the young people came home from school and needed a snack or between meals. Staff attempted to place restrictions on the intake of products perceived to be unhealthy by not serving the product in question:

We also drink a lot of juice, but there is also often milk and water. I usually put out water and milk and they can get juice themselves if they want it. Trying to make sure that they do not have to drink juice all the time. (staff informant 7)

Institutional guidelines were perceived by many of the staff informants to induce ‘cooking from scratch’ to the extent possible. In some instances, this food was unfamiliar to the young people since, according to the staff, ready-made products had been frequently used in their previous homes, and some had been used to eating ‘on the go’. There was some disaccord detected among staff informants with regard to holding the line and only serving the food that was on the menu, while some young people were encouraged to cook something else for themselves if they did not like what was served. Meanwhile, the view of the majority of staff seemed to be that they would not let anyone starve but would not cook anything else for dinner either:

If they don’t like the food, they simply have to eat bread instead … that’s at least the rule we try to have. But they can also buy food of their own, so they can go and buy something with their own pocket money. So that happens too … (staff informant 11)

Interestingly, one of the staff reflected on a time where a soft approach was no longer possible due to a very unhealthy eating pattern in one of the residing youths (e.g. ‘consuming pure sugar from the sugar bowl’). This seemed to have been uncomfortable, and the staff respondent appeared relieved that it was not an everyday occurrence:

(…) for me, it is nice to let go of that (…) that you must have control over the food. (staff informant 7)

#### Prepare

Food preparation was an everyday activity. All staff informants said that the young people had every chance to participate in food preparation if they wanted to. This finding was also corroborated among the youths. However, dinner was usually prepared during daytime or early afternoon on weekdays, and the young people were often absent or tired after school. In general, staff informants expressed understanding for the young persons’ desire to relax instead of helping out with dinner. Some youths, however, were viewed by some staff informants as ‘having an interest in cooking’ and would participate willingly. For instance, one youth informant expressed that she would use this opportunity to engage and use her agency to get the food she wanted:

I like sometimes to help, just to have food that is tasty. You know what I mean? If I know that there is someone that is not good at making food, I just help sometimes. (youth informant 8)

Meanwhile, another young person expressed how the food offered by the institution was tempting to accept since it would save her from the extra effort that cooking entailed:

I actually make my own food all the time. And I can make my own dinner. Ehh, but … like, if someone is offering me food, why should I bother to cook? (laughing). (youth informant 1)

To motivate the young people to participate in preparing food, setting the table, and cleaning up after a meal, some institutions had chosen to give pocket money as a reward. Meanwhile, one staff informant said that in their institution, they did not want to link pocket money to food because some young people could have a problematic relationship with food, and for them, the system would be unfair. Another staff informant was concerned that food-related activities were something that the youths had to handle themselves in the long run, and using pocket money as an incentive for participation could interfere with internal motivation:

(…) and also getting paid to do it is a bit uncertain if it… about getting paid … in a way you don’t get paid once you live by yourself. (staff informant 11)

The ‘window of opportunity’ for the staff to discuss healthy food and demonstrate food preparation with the young people was mentioned by several staff informants to be dinnertime on weekends, when the setting was more relaxed. Even though the young people would not actively participate, they would commonly sit and observe as dinner was prepared. The importance of contributing to the young people viewing cooking as enjoyable was also emphasized by a few staff informants. The staff further referred to food preparation as an opportunity for observational learning:

Eh … personally at least, from time to time I experience that youths choose to sit by the kitchen table while I cook. So that they are close by, so if they don’t (…) at least that is how I view things, with regards to environmental therapy, that even if they do not physically participate in cooking, they observe, all, they observe it, how you, what foods you take out, you may chop up and that the processes, it is, you get the smell, you kind of get all the other elements by being in the kitchen. (Staff informant 11)

The natural setting around cooking also seemed to provide opportunity for the youths to engage in good conversations and address matters they would otherwise not share:

Because it, both eh … the feeling of unity around cooking food, that they simply let go, a few times I have experienced it … that youths, you see them preoccupied with something, you see that they have something to talk about which they are not ready for, that only sitting in the kitchen while you are cooking (…) you see that they start to relax more and more (…). The environmental therapy around meal preparation takes place in the kitchen. (staff informant 13)

#### Eat

Eating relates not only to the ability to evaluate the connection between food and health but also to the participation and appreciation of the social aspects of eating. Participating in shared meals was not a requirement, and the young people always had the opportunity to walk away. While the dinner meal was mentioned by most staff and some young persons as a point of nice conversation and socialization, it could quickly become a difficult setting for some. Social anxiety or the young person’s relationship with either the other residents or staff could make mealtimes seem like an unsafe area. Furthermore, two of the staff informants reported that in some cases, it was necessary to separate the young people based on the relationship they had. As one staff informant said:

(…) it is good that more than one adult is present … if the mood changes, suddenly there may be a fight or that they … that it becomes, you need to watch a bit the social (…) it may become a bit disagreeable and then you must try to guide them a bit and … discretely turn the conversation to something else, and then it is nice to be more adults present to … stand united if something happens. (staff informant 11)

Strained meal situations and not getting along were also reflected upon by some youth informants:

Sometimes it is very often one or two people who talk only about themselves and what they want to talk about. I get a little tired of it, so I just go back to my room or go into the living room or something. (youth informant 8)

### Autonomy and restrictions

In addition to the themes related to food literacy, by reading and analyzing the interviews, new inductive themes emerged, such as ‘autonomy and restrictions’ or ‘the need for a soft approach’.

From the staff’s perspective, youth participation in meal preparation and shared meals, both important elements in the food literacy model, seemed to be an issue closely related to autonomy. Some referred to dinner as a meal where the young people knew that their presence was expected. One staff informant explained that they would go knocking on doors before dinner was served, hoping to induce participation. However, despite these efforts, many residents chose not to attend shared meals, which seemed to frustrate some staff (since they could not always comprehend the reason) while, at the same time, recognizing that attendance could not be forced:

In a way, we cannot physically force them to come sit by the table, eh … . (staff informant 2)

Similarly, another staff informant expressed that demanding that the young people eat together felt like stepping over their boundaries. Still, a long meal where all young people participated seemed to be cherished by many staff. The youths also reflected on how participation in common meals had been practiced differently in the institutions they were familiar with. One youth informant felt that if residents were having a bad day and wanted to be alone, they should be allowed to eat by themselves:

If you have a bad day and you just want to be left alone, then you must have the opportunity to eat by yourself. (youth informant 1)

One staff informant expressed concern that some young persons kept the consumed soda in their own room every day, but at the same time, they added that this was not as big a challenge as some other issues the youths struggled with. Forcing participation in food-related activities was viewed by some as a potential source of conflict, which the staff were trying to avoid. Unhealthy meal patterns among the young people, such as eating during the night and skipping breakfast, were further recognized as a problem by some staff informants, but at the same time, they expressed understanding and came up with potential reasons for the young person’s choices (e.g. eating disorders or having a bad day). Denying the young persons’ food was also viewed as problematic:

(…) that food is eaten at nighttime, eh, we try to limit as much as possible, that you eh … well … But again, it’s a (…) difficult too, you know, the issue of denying, denying someone food, you, like, can’t deny someone food either. (staff informant 10)

Another area closely linked to youth autonomy was pocket money. Several staff mentioned that the food the young people consumed with friends was often unhealthy options, such as fast food. This was paid for with the youths’ own money and, thus, was described by several staff informants as something they could not or should not influence. One staff informant explained that candy purchased for the institution was locked away and reflected on how this would not be the case in a ‘normal home’. Candy purchased for pocket money was not to be consumed in the common area but appeared in most institutions to be eaten freely in the young persons’ rooms. Furthermore, one staff informant remembered that at one point, some candy a youth had bought with their own money had been taken from them, which was considered a breach of trust.

### The need for a soft approach

Several staff informants expressed concern that the young people residing in childcare institutions more commonly have problematic relations with food, such as eating disorders. For this reason, they would tread carefully when addressing the topic of healthy food:

So in a sense contribute to giving them healthy habits, a healthy relationship with food without contributing to, like, one or the other. (staff informant 12)

Interestingly, a healthy meal pattern and intake of healthy foods were also viewed as important to stabilize blood sugar levels among the young people, which the staff related to reducing the risk of conflicts. Furthermore, some staff informants felt that food or meals were sometimes the ‘engine’ of a conflict that was actually due to the youth having a bad day for some other reason. In some instances, the staff expressed that they instinctively knew when a young person was having a bad day, in which case, they would back off and not push participation in order to avoid conflicts:

… and then you have some who are in a way (…), so if we talk about what a healthy diet is for instance … some in a way what do you think that I am fat? Well … so it comes out like that, right, and others have so high triggers that … well … the goal is to get food in them to lower … eh … to lower their adrenalin so they … don’t explode right, and then, what you feed them is less important, you just need to get food in them (…). So that, that is how it is from time to time, you have such different needs, so it is difficult to have common rules because all have different needs. (Staff informant 13)

Although all staff respondents appreciated the importance of healthy food for the young people’s physical and mental well-being, one staff informant expressed that ‘a soft approach’ was sometimes chosen due to the young person’s background. This was also related to demanding youth participation:

… many of these kids in a way can’t ever have had anyone demanding that they help in a way to cook, so that they do not know what it entails. (staff informant 2)

All in all, the general impression given by all staff informants was that they should provide opportunities to improve food-related skills among the young people without adding too much pressure or force. As one staff informant said:

I don’t know, it varies a lot which youths are here and who is interested and … so, we can’t … I am not very much in favor of pushing them into something they absolutely do not feel like doing because then you get nowhere, and they won’t learn anything either. (staff informant 7)

In line with this, another staff respondent voiced that while the significance of diet for health and well-being was well incorporated among the staff, the challenge was making the youths realize this themselves.

## Discussion

The purpose of our study was to explore how food literacy is transmitted to young persons residing in childcare institutions through involvement and participation in food-related activities. Participation by young persons in planning menus, shopping for groceries, cooking, and common meals was encouraged by staff in the childcare institutions. This was also reflected among the youths. We further found that the institutional context provided both opportunities and limitations for the staff to transmit food literacy to the young people, and that different potentially conflicting aims tend to emerge. Food was mentioned as having symbolic meaning as a token of care and shared experiences, where the young people observing the staff cooking from scratch and participation in common meals were seen as important arenas for socialization and expanding relationships between young people and staff. The staff tended to prefer what we may call a soft approach to encouraging participation in food-related activities due to the young persons being perceived as vulnerable and a focus by staff on youth autonomy. Demanding that the youths take responsibility for food-related tasks or participation in common meals was perceived to increase the risk of conflict.

An important finding in our study was how the institutional context provided both opportunities and limitations for transmitting food literacy. Importantly, a childcare institution is at the intersection between homely and public spheres, where staff work to provide young people with a sense of ‘being a family’ within the limitations of their institutional role (24). Making good use of the ‘windows of opportunity’ to develop food literacy and laying the groundwork for the transfer of knowledge that would benefit the young people in adulthood (8, 13) were perceived by staff as part of both these spheres. Youths with different backgrounds, time schedules, and experiences with institutions living under the same roof were challenging, since common rules regarding participation in food-related activities and food intake could not be applied. Living in an institution may also delay the young person’s sense of urgency with regard to learning food literacy, since while a gradual increase in food-related competencies was an aim expressed by the staff, the institution would also provide meals, which might decrease motivation among young people to manage on their own.

Engagement in all food-related activities seemed to increase on weekend days when the young people were allowed to add their preferred meals to the menu. At the same time, healthy food choices, such as fish and vegetables, were often not preferred. Our study, therefore, implies a potential dilemma for staff where transmitting food literacy is sometimes at odds with consuming healthier diets, and where serving unhealthy food to children and young people may incite a feeling of not being a good caregiver (28). Since food choices such as fish and vegetables were often not preferred, they could generate a bad atmosphere or children skipping meals together. Similar dilemmas also emerged in a previous study conducted in Norway, showing variations in how childcare institutions balance structure (e.g. healthy food choices for all residents and demands for meal participation) versus flexibility (22). Implementing measures to increase the healthiness of preferred meals and making meals with vegetables and fish inviting and palatable accompanied by a principal discussion about the relative importance of participation in relation to healthiness and institutional guidelines seem warranted.

Views of food and meals as symbols or rituals were touched upon in various ways during the interviews. Comparable to our findings, a study by Punch et al. conducted in childcare institutions in Scotland found that food was viewed in many instances as a ‘material expression of something else’. This something else could be creating a home, implementing structure and routines, helping with transitions, care and control, negotiating relationships, and inciting autonomy (23). In our study, viewing food as a form of care was reflected by staff preparing school lunches, albeit recognizing that it was unnecessary based on the young person’s age. This corroborates findings in previous studies showing that for youths who experience parents being absent, food and meals are arenas for staff to show care and consideration, love and support, and strong relations (29, 30). Common meals were viewed as a proxy for socialization and a homely atmosphere among our informants, which has also been shown previously (24). Furthermore, cooking was expressed to be a door-opener toward building relationships with the young people. Their opening up was believed to be due to a natural and relaxed setting and time together spent on a common activity. In this regard, food learning should be viewed not only as the transmission of information but also involving emotional and social experiences (7). Finally, meals were viewed by many as ‘high risk situations’. The contrast between the family meal ideal and praxis also emerged in other studies of domestic dinners (31). Interestingly, this was also reflected in the study from Scotland, where staff in childcare institutions referred to meals as ‘the best and worst times of the day’ (23).

The difficulties in gathering people for common meals, which is a requirement for learning how to eat in a social way as part of the food literacy framework (10), could also be due to the fact that, as staff in this study stated, some residents brought with them the habit of consuming food on the go and may have had fewer opportunities for participating in food-related activities in their families compared to young people in general. While recognizing the importance of transmitting food literacy to the young people, concern about psychological health and eating disorders was a main barrier to this transmission, where evidence of unhealthy food consumption in the young persons’ own rooms and distorted eating behaviors (e.g. night eating) were reflected in the interviews. There is a higher prevalence of psychological challenges among young people in childcare institutions compared to the general population (32). These youths also have a higher risk of overweight and obesity, which may partly be caused by neglect (33, 34). Furthermore, young people in childcare services may have had food-related challenges in their childhood, which negatively influences their motivation and ability to self-regulate. For instance, experiencing inadequate or unstable access to food growing up may cause overeating or hording when food is readily available (34).

Alongside concerns about the young persons’ psychological health, youth autonomy and a fear of inciting conflicts made the staff take what we can define as a soft approach to transmitting food literacy and promoting a healthier diet (e.g. allowing the youths to choose when and where to eat and serving milk or water instead of juice). While a good system for childcare allows for user involvement and ensuring that children’s or young people’s opinions are considered to the extent possible (35), caring sometimes also entails exercising control over residents when needed (23). Interestingly, in the study in childcare institutions in Scotland, unfair or negative control was perceived by residents when staff exerted pressure into changing their diets (23), which was also reflected in this study, where a soft approach was preferred by the young people. The soft approach also related to participation in cooking, where the staff seemed content if the young persons would sit and observe as they prepared dinner, hoping to incite curiosity and perhaps, in the long run, change cooking and eating habits. This notion is to some extent supported by a study among Canadian college students, showing that food-related learning had occurred primarily through observation of their parents’ food habits. At the same time, observation will not improve cooking self-efficacy, which is a barrier to food literacy mentioned in previous studies (11).

Adolescence is marked by a transition from mainly parental-controlled eating to more peer-influence and self-reliance regarding food decisions (36). During this life stage, young people tend to replace food from home with food preferences shared by peers (37), where peer influence over food intake seems to be strongest at 15–19 years (6). A positive association between the level of autonomy and snack purchasing has been identified (7), which was reflected in our data where eating with friends often led to unhealthier intake of takeaway and fast foods than if food were consumed in the institution. Young people tend to exert autonomy in food choices through spending (36). In light of this, pocket money was found to be a tool to incite a feeling of autonomy, since staff overall agreed that they should not meddle with how this money was spent, despite realizing that it would often be spent on unhealthy food or candy. The dual vulnerability to poor food literacy and unhealthy eating behaviors emphasizes the importance of creating food-learning opportunities prior to transition into adult life (14, 38) in this group of young people.

This study had several strengths. First, few studies have investigated the transmission of food literacy in this age group in childcare institutions in Norway and, to the best of our knowledge, internationally. We interviewed both staff and residents, which provides a more comprehensive understanding – particularly in relation to barriers – to transmitting food literacy in childcare institutions. Individual interviews allowed for openness probably beyond what would be expected in focus group interviews with colleagues. Furthermore, the individuals conducting the interviews were public health nutritionists with a thorough understanding of food literacy and dietary challenges among young people, which likely improved the chances of relevant follow-up questions being posed. Meanwhile, given the professional background of the researchers, a social desirability bias may occur with respondents trying to put themselves or their institution in a favorable light. Interviewing both staff and youths may have contributed to reducing this potential bias, as different perspectives were gathered. Thirteen staff members and eight young people were interviewed, representing all institutions but one.

A limitation that may have affected data saturation is that the recruitment of young persons went through adults, and it may be the case that those having better relationships with the adults were more willing to participate. However, this approach was chosen to protect the privacy of the youths living in the institutions and to respect ethical guidelines. Another limitation is that few men were interviewed; however, this is representative for the gender balance in Norwegian childcare services (39). Finally, due to the COVID-19 pandemic, the interviews were conducted using Microsoft Teams, which may have influenced the relationship between the researcher and the participants and, hence, the quality of the data. However, during and after the pandemic, the use of Teams in research and everyday activities has become mainstream, and recent studies seem to suggest that – particularly for studies like ours – it may not have an impact on the quality of the interviews (40). A strength of this study is that findings from the analysis of the interviews were presented at different stages to a reference group consisting of three staff members working in the institutions and two young persons. This provided us with important feedback for interpretations and further analysis of the data.

## Conclusions

This study showed that food and meals are an important part of the relationship between caretakers and young people living in childcare institutions, and that living in the institution constituted an opportunity for improving food-related skills and dietary habits before adulthood. However, opportunities were often not transformed into action due to various reasons such as an emphasis on youth autonomy, the risk of getting into conflicts, and concerns about being insensitive with regards to eating disorders. Initiatives aimed at improving food literacy in childcare institutions are recommended. For instance, encouraging social gatherings in the common areas when food is prepared on weekends may provide an opportunity to engage young people in discussions about food while demonstrating cooking in practice.
